# Curcumin Protects Diabetic Mice against Isoproterenol-Induced Myocardial Infarction by Modulating CB2 Cannabinoid Receptors

**DOI:** 10.3390/life12050624

**Published:** 2022-04-22

**Authors:** Harshal D. Pawar, Umesh B. Mahajan, Kartik T. Nakhate, Yogeeta O. Agrawal, Chandragouda R. Patil, M. F. Nagoor Meeran, Charu Sharma, Shreesh Ojha, Sameer N. Goyal

**Affiliations:** 1 Department of Pharmacology, R. C. Patel Institute of Pharmaceutical Education and Research, Shirpur 425405, India; hpawar542@gmail.com (H.D.P.); umeshmahajan41@gmail.com (U.B.M.); crpatil@rcpatelpharmacy.co.in (C.R.P.); 2 Shri Vile Parle Kelavani Mandal’s Institute of Pharmacy, Dhule 424001, India; kartik.nakhate@svkm.ac.in (K.T.N.); yogeeta.goyal@svkm.ac.in (Y.O.A.); 3 Department of Pharmacology and Therapeutics, College of Medicine and Health Sciences, United Arab Emirates University, Al Ain P.O. Box 15551, United Arab Emirates; nagoormeeran1985@uaeu.ac.ae (M.F.N.M.); shreeshojha@uaeu.ac.ae (S.O.); 4 Department of Internal Medicine, College of Medicine and Health Sciences, United Arab Emirates University, Al Ain P.O. Box 15551, United Arab Emirates; charusharma@uaeu.ac.ae; 5 Zayed Bin Sultan Center for Health Sciences, United Arab Emirates University, Al Ain P.O. Box 15551, United Arab Emirates

**Keywords:** curcumin, CB2 receptor, myocardial infarction, oxidative stress, diabetes

## Abstract

Molecular docking revealed curcumin as a potent CB2 cannabinoid receptor (CB2R) agonist. Since CB2R is involved in cardioprotective functions, we explored its role in ameliorative actions of curcumin against myocardial damage triggered by isoproterenol in diabetic animals. Mice were kept on a high-fat diet (HFD) throughout the experiment (30 days). Following 7 days of HFD feeding, streptozotocin was administered (150 mg/kg, intraperitoneal) to induce diabetes. From day 11 to 30, diabetic mice received either curcumin (100 or 200 mg/kg/day, oral), CB2R antagonist AM630 (1 mg/kg/day, intraperitoneal) or both, with concurrent isoproterenol (150 mg/kg, subcutaneous) administration on day 28 and 29. Diabetic mice with myocardial infarction showed an altered hemodynamic pattern and lipid profile, reduced injury markers, antioxidants with increased lipid peroxidation in the myocardium, and elevated glucose and liver enzymes in the blood. Moreover, an increased pro-inflammatory markers, histological severity, myonecrosis, and edema were observed. Curcumin compensated for hemodynamic fluctuations, restored biochemical markers, preserved antioxidant capacity, decreased cytokines levels, and restored cardiac functionality. However, the AM630 pre-treatment attenuated the effects of curcumin. The data suggest the involvement of CB2R in the actions of curcumin such as in the prevention of myocardial stress and in the improvement of the normal status of the myocardial membrane associated with diabetes.

## 1. Introduction

The risk of cardiovascular morbidity and mortality in diabetic patients is 2- to 4-fold higher than non-diabetic persons. [1]. Indeed, in patients of diabetes mellitus, myocardial infarction (MI) is the foremost cause of mortality. The incidence of MI in diabetic conditions increases depending on the number of comorbidities such as hyperglycemia, dyslipidemia, oxidative stress, and inflammatory conditions [2]. Interestingly, the pathogenesis of diabetes and MI involves the secretion of inflammatory mediators and the excessive formation of reactive oxygen species (ROS) [3,4]. In cardiac cells, oxidative stress is exacerbated by chronic hyperglycemia which is associated with diabetes, and intensifies the depletion of the myocardial membrane through lipid peroxidation and the inflammatory cytokinetic response [5]. Moreover, the intake of calorie-rich high-fat diet (HFD) can lead to the over-deposition of adipocytes-generating adipokines that develop insulin resistance and systemic inflammation [6].

The endocannabinoid system has been widely studied as a potential target for diseases associated with tissue injury and inflammation of the cardiovascular system. Cannabinoid receptors type-2 (CB2R) are ubiquitously present in many tissues including cardiac myocytes [7]. High inducibility is an interesting property of CB2R as the injury or inflammatory process can increase their mRNA expression as much as 100-fold [8]. Therefore, the activation of CB2R seems to have a therapeutic value in conditions such as MI. This line of argument is supported by the fact that the deletion of CB2R deteriorates MI [9]. Emerging pieces of evidence also suggest the involvement of CB2R in cardioprotective functions. While CB2R agonists reduce the infarct size associated with ischemia or reperfusion, the blockading of CB2R abolishes the cardioprotective action of endocannabinoids [7,10]. Attempts have been made to identify the naturally occurring ligands such as phytoconstituents that engage CB2R. Curcumin, a polyphenol derived from perennial plant *Curcuma longa*, aids in managing oxidative stress, inflammation, and hyperlipidemia [11,12,13]. Moreover, curcumin improves systolic dysfunction and prevents cardiac remodeling after MI [14]. Despite these findings, the involvement of CB2R in the cardioprotective effects of curcumin remains unexplored.

Against this background, our aim was to assess whether curcumin, as a CB2R agonist, can alleviate the MI in diabetic mice on HFD by improving cardiac functionality, biochemical injury markers and tissue architecture. We used a mouse model of streptozotocin (STZ)-induced diabetes combined with isoproterenol (ISO) administration to generate pathophysiological and morphological changes similar to those in diabetic patients with MI [15]. STZ develops the diabetic condition by inducing the destruction of pancreatic beta cells [16]. ISO (nonselective beta-adrenoceptor agonist) causes MI by generating highly cytotoxic free radicals, inflammatory mediators, cytosolic Ca^++^ overload, and apoptosis [17]. To evaluate the intensity of the agonistic behavior of curcumin at CB2R, a molecular docking study was undertaken.

## 2. Materials and Methods

### 2.1. Animals

Adult male Swiss albino mice (20–25 g) were used. They were kept under natural light and a dark cycle, at constant room temperature (25 ± 5 °C) and humidity (55 ± 5%). The animals were fed with a pelleted chow diet (Nutrivet Life Sciences, Pune, India) or HFD (Research Diet Inc., New Brunswick, NJ, USA) and water ad libitum. The experimental protocols were approved by Institutional Animal Ethics Committee (Approval No. IAEC/CPCSEA/RCPIPER/2018-19/13) of RCPIPER, Shirpur, Maharashtra, India.

### 2.2. Chemicals

Curcumin, AM630, STZ, and ISO were procured from Sigma-Aldrich, Saint Louis, MO, USA. Curcumin was dispersed in 0.5% *w*/*v* carboxymethyl cellulose prior to administration. The kits for the determination of creatine kinase-MB (CK-MB) isoenzyme, lactate dehydrogenase (LDH), serum transaminase, total cholesterol (TC), triglycerides (TG), low-density lipoprotein cholesterol (LDL-C) and high-density lipoprotein cholesterol (HDL-C) were procured from ERBA Diagnostics, Germany. The mice ELISA kit for tumor necrosis factor (TNF-α), interleukin-6 (IL-6) and IL-1β was purchased from eBioscience, San Diego, CA, USA. Other reagents of analytical grade were procured from local suppliers.

### 2.3. Induction of Experimental Diabetes

Animals were randomly divided into six groups. Throughout the period of experimentation (30 days), the five groups of mice were kept on HFD ad libitum. Diabetes was induced in mice on HFD using a single injection of STZ (150 mg/kg) via an intraperitoneal (i.p.) route on the 7th day of the study. In parallel, a control group of animals was kept on a pelleted chow diet and did not receive any treatment. The solution of STZ was prepared in ice-cold 0.1 M cold acidic (pH 4.5) citrate buffer and instantly administered. On the 10th day, STZ-treated mice with fasting blood glucose levels of higher than 250 mg/dL were considered as diabetic and used in the following studies.

### 2.4. Induction of Experimental Myocardial Infarction

ISO (150 mg/kg) was administered via a subcutaneous (s.c.) route to diabetic mice for two consecutive days (day 28 and 29) for the induction of experimental MI.

### 2.5. Experimental Protocols

A normal control and diabetic groups (*n* = 6 per group) received treatments from the 11th day to 30th day of the study as mentioned below.

*Group I—Normal*

Mice were orally administered 0.5% carboxymethylcellulose throughout the protocol (day 11 to 30), and on day 28 and 29, they were administered with saline (0.3 mL, s.c.) at 24 h interval.

*Group II—HFD + STZ + ISO*

In mice fed with HFD, STZ (150 mg/kg, i.p.) was administered on day 7. Thereafter, ISO (150 mg/kg, s.c.) was administered on day 28 and 29 at 24 h intervals.

*Group III—HFD + STZ + ISO + Curcumin*

HFD-fed diabetic mice were treated with curcumin (100 mg/kg/day, p.o.) from day 11 to 30, and ISO (150 mg/kg, s.c.) was administered concurrently on day 28 and 29.

*Group IV—HFD + STZ + ISO + Curcumin*

HFD-fed diabetic mice were treated with curcumin (200 mg/kg/day, p.o.) from day 11 to 30, and ISO (150 mg/kg, s.c.) was administered concurrently on day 28 and 29.

*Group V—HFD + STZ + ISO + AM630 + Curcumin*

HFD-fed diabetic mice were treated with AM630 (1 mg/kg/day, i.p.) 1 h prior to curcumin (200 mg/kg/day, p.o.) from day 11 to 30, and ISO (150 mg/kg, s.c.) was administered concurrently on day 28 and 29.

*Group VI—HFD + STZ + ISO + AM630*

HFD-fed diabetic mice were treated with AM630 (1 mg/kg/day, i.p.) from day 11 to 30 and ISO (150 mg/kg, s.c.) was administered concurrently on day 28 and 29.

Throughout the experimentation, the changes in the food intake and body weight, as well as the mortality rate of different groups of animals were monitored.

### 2.6. Experimental Parameters

A hemodynamic assessment was performed using the procedure described in our previous study [18]. Experimental animals were kept in a supine position on a wooden board and an electrocardiogram (ECG) was recorded continuously using lead-III skin electrodes, with II electrodes towards the heart on the right and left forelimbs and the neutral 3^rd^ electrode on the hind limb facing the heart; these electrodes were connected to the data acquisition system (Power Lab, AD Instruments, Sydney, Australia).

### 2.7. Biochemical Parameters

Myocardial tissue homogenate (10%) was prepared in an ice-chilled phosphate buffer (50 mM, pH 7.4), and using aliquot, the amount of cardiac injury marker CK-MB and LDH was estimated as per manufacturer’s instructions. Similarly, the glucose level, lipid profile, glutamic oxaloacetic transaminase (SGOT) and glutamic pyruvic transaminase (SGPT) were estimated in serum samples.

### 2.8. Estimation of Oxidative Stress

The oxidative stress was assessed by analyzing the contents of malondialdehyde (MDA), reduced glutathione (GSH), catalase (CAT) and the activity of superoxide dismutase (SOD) [17,19,20].

### 2.9. Estimation of Inflammatory Cytokines

Pro-inflammatory cytokines such as TNF-α, IL-6 and IL-1β were measured using ELISA kits as per the manufacturer’s instructions. The color intensity was measured with a Synergy H1M multimode microplate reader, BioTek, India. The activity of TNF-α, IL-6 and IL-1β was expressed as pg/mL.

### 2.10. Light Microscopy Examination

The serial sections of heart tissue were obtained using a microtome. The sections were stained with hematoxylin and eosin, observed under the microscope, and digital images of stained tissue were captured using Motic image software 2.0. The pathologist involved in the examination and microscopy was blind to the treatment groups.

### 2.11. Molecular Docking Study

The protein data bank (RCSB) was used to obtain a G protein coupled CB2R (6KPC) crystal structure. The ChemDraw Professional 16.0 software was used to design a 2D structure of the ligands, i.e., curcumin and a selective CB2R agonist JWH-133. The molecular mechanics optimization of the 2D structures of the ligands into the 3D structures was performed using the Chem 3D 16.0 application of the same software. Further, for molecular docking, the Argus Lab 4.0.1 software was used. The grid with dimension X = 37.5236, Y = 22.4981, and Z = 20.6868 Å were assigned to cover the entire 3-dimensional active site of CB2R. Dynamic molecular docking was carried out on the active site of protein (CB2R). The binding energy was retrieved, and the CB2R–curcumin interaction was visualized using Discovery Studio 2016 software [21,22].

### 2.12. Statistical Analysis

The data of each group were represented as mean ± standard error mean (SEM). A statistical analysis was performed using the analysis of variance (ANOVA) followed by a post hoc Bonferroni’s multiple comparison test using Graph Pad Prism software (Version 6.0). A *p* value of less than 0.05 (*p* < 0.05) was considered as statistically significant.

## 3. Results

A mortality rate of 8% was observed due to MI. Variations in the body weight of diabetic mice were observed when compared to their basal readings.

### 3.1. Effect of Curcumin on Body Weight and Food Intake in HFD Fed Myocardial Infarcted Diabetic Mice

**Figure 1:**
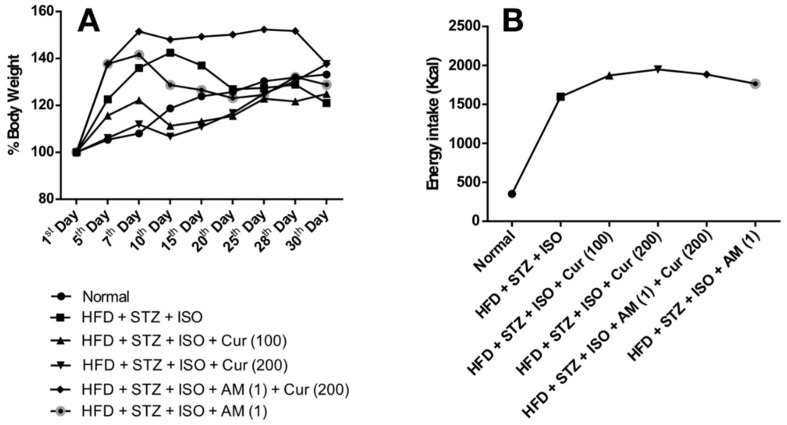
Effect of curcumin on body weight and food intake in HFD-fed myocardial infarcted diabetic mice. (**A**) Percentage change in body weight; (**B**) Food intake (Energy). The data were expressed as mean ± SEM, and analyzed by one-way analysis of variance (ANOVA) and two-way ANOVA, respectively, followed by Bonferroni’s post hoc test. AM: AM630; Cur: Curcumin; HFD: High-fat diet; ISO: Isoproterenol; STZ: Streptozotocin.

HFD is well-known to induce changes in the lipid metabolism and leads to an increase in metabolic abnormalities. It is effective in uplifting the body weight as well as the abdominal obesity in mice. After the induction of diabetes by STZ, the body weight reduced significantly (*p* < 0.05) in all groups of animals except in the normal group that did not receive STZ. Curcumin treatment maintained the body weight significantly (*p* < 0.05). The HFD + STZ + ISO group showed a significant (*p* < 0.05) reduction in the total Kcal consumption of HFD as compared to HFD + STZ + ISO + Curcumin (100 and 200). In the HFD + STZ + ISO group, a significant (*p* < 0.05) increase in the total Kcal consumption of HFD was observed as compared to normal mice (Figure 1).

### 3.2. Effect of Curcumin on Electrocardiogram in HFD Fed Myocardial Infarcted Diabetic Mice

**Figure 2:**
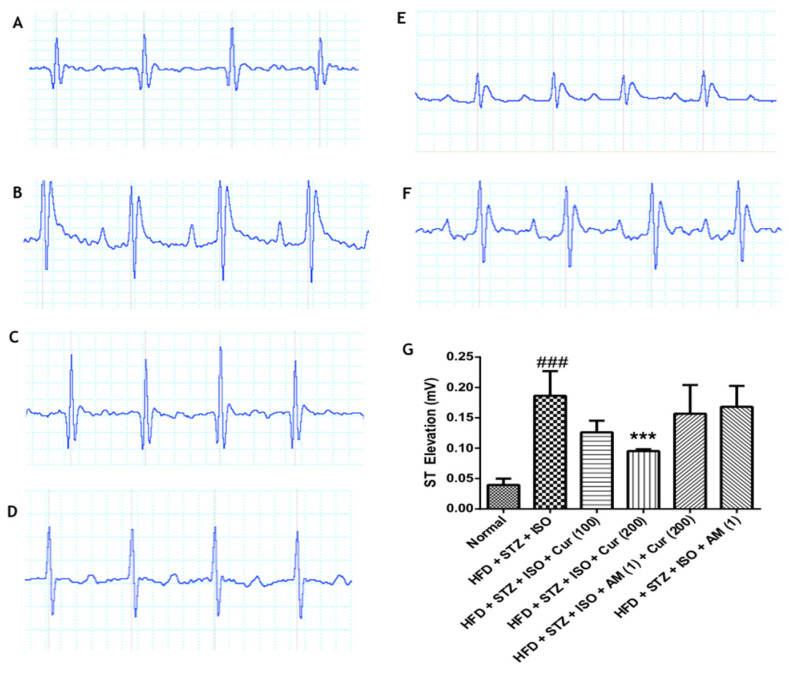
Curcumin improved the electrocardiogram in HFD-fed myocardial infarcted diabetic mice. (**A**) Normal; (**B**) HFD + STZ + ISO; (**C**) HFD + STZ + ISO + Cur (100); (**D**) HFD + STZ + ISO + Cur (200); (**E**) HFD + STZ + ISO + AM (1) + Cur (200); (**F**) HFD + STZ + ISO + AM (1); (**G**) ST height. The data were expressed as mean ± SEM, and analyzed by a one-way analysis of variance (ANOVA) followed by Bonferroni’s post hoc test. ^###^ *p* < 0.001 vs. normal; *** *p* < 0.001 vs. HFD + STZ + ISO. AM: AM630; Cur: Curcumin; HFD: High-fat diet; ISO: Isoproterenol; STZ: Streptozotocin.

Changes and alterations in the ECG waveforms and its pattern such as the ST segment elevation and increased ST height indicate MI. This was observed in the HFD diabetic mice administered with ISO (*p* < 0.01). The administration of curcumin (200 mg/kg) prevented ST segment elevation and decreased ST height as compared to HFD + STZ + ISO (*p* < 0.001). However, AM630 significantly inhibited the effects of curcumin, which indicates the involvement of CB2R in the cardioprotective effects of curcumin (Figure 2).

### 3.3. Effect of Curcumin on Blood Glucose Level and Lipid Profile in HFD Fed Myocardial Infarcted Diabetic Mice

A significant hyperglycemia (*p* < 0.001) was observed in the HFD + STZ + ISO-treated animals as compared to normal mice. Treatment with curcumin (200 mg/kg) significantly (*p* < 0.01) restored the level of glucose as compared to the HFD + STZ + ISO group. However, AM630 significantly blocked the activity of curcumin. The lipid profile was altered and was found to be increased in the HFD + STZ + ISO group as compared to normal mice. Curcumin (200 mg/kg) significantly (*p* < 0.001) normalized the lipid profile viz., TC, TG, and LDL-C, and increased HDL-C (Table 1).

### 3.4. Effect of Curcumin on Cardiac and Liver Injury Markers in HFD Fed Myocardial Infarcted Diabetic Mice

**Figure 3:**
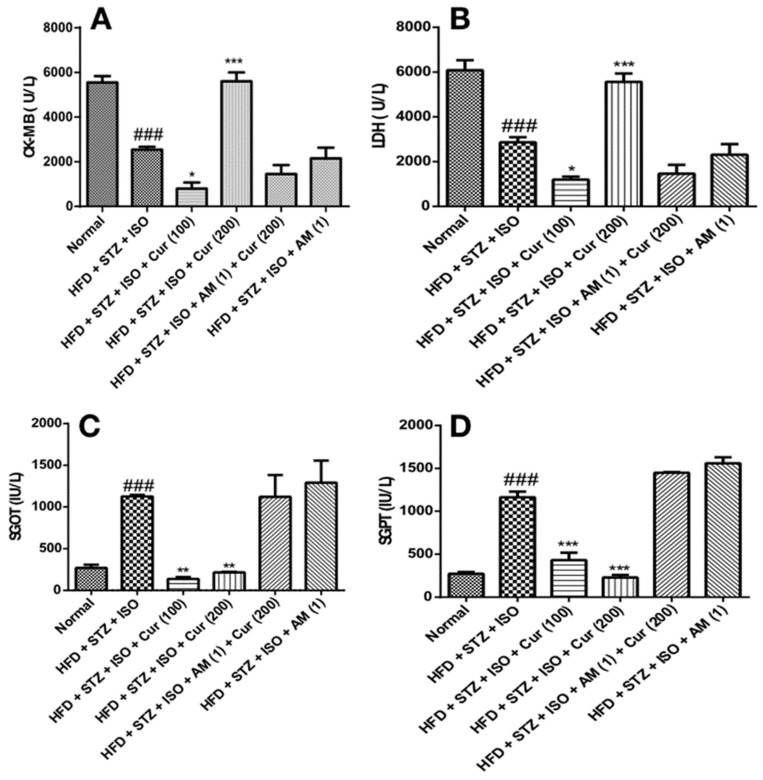
Curcumin attenuated the cardiac integrity and liver injury markers in HFD-fed myocardial infarcted diabetic mice. (**A**) CK-MB; (**B**) LDH; (**C**) SGOT; (**D**) SGPT. The data were expressed as mean ± SEM and analyzed using one-way analysis of variance (ANOVA) followed by Bonferroni’s post hoc test. ^###^ *p* < 0.001 vs. normal; * *p* < 0.05, ** *p* < 0.01, *** *p* < 0.001 vs. HFD + STZ + ISO. AM: AM630; CK-MB: Creatine kinase-MB; Cur: Curcumin; HFD: High-fat diet; ISO: Isoproterenol; LDH: Lactate dehydrogenase; SGOT: Serum glutamic-oxalacetic transaminase; SGPT: Serum glutamic pyruvic transaminase; STZ: Streptozotocin.

The levels of cardiac markers (CK-MB and LDH) were considerably decreased (*p* < 0.001) in the HFD + STZ + ISO-treated mice. Curcumin at a dose of 200 mg/kg significantly increased (*p* < 0.001) the contents of CK-MB and LDH in myocardium, thereby indicating the integrity of the cardiac membrane. However, the concomitant prior administration of AM630 attenuated the protective effects of curcumin. Additionally, a significant increase in the levels of SGOT and SGPT (*p* < 0.001) was observed in the HFD + STZ + ISO-treated mice. Curcumin suppressed the level of SGOT and SGPT in serum (*p* < 0.05) compared to the HFD + STZ + ISO-treated animals. This effect of curcumin, however, was attenuated by AM630 (Figure 3).

### 3.5. Effect of Curcumin on Oxidative Stress in HFD Fed Myocardial Infarcted Diabetic Mice

**Figure 4:**
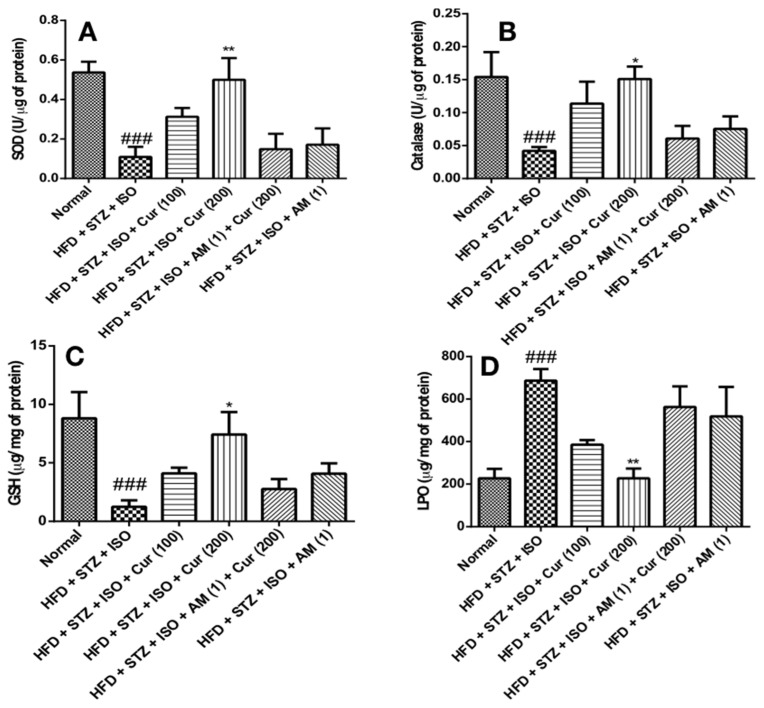
Curcumin restored the oxidative stress in HFD-fed myocardial infarcted diabetic mice. (**A**) SOD; (**B**) Catalase.; (**C**) GSH; (**D**) LPO. The data were expressed as mean ± SEM and analyzed by using one-way analysis of variance (ANOVA) followed by Bonferroni’s post hoc test. ^###^ *p* < 0.001 vs. normal; * *p* < 0.01, ** *p* < 0.001 vs. HFD + STZ + ISO. AM: AM630; Cur: Curcumin; GSH: Reduced glutathione; HFD: High-fat diet; ISO: Isoproterenol; LPO: Lipid peroxidation; SOD: Superoxide dismutase; STZ: Streptozotocin.

A significant depletion (*p* < 0.001) in the contents of SOD, CAT and GSH was noted in the group administered with HFD + STZ + ISO compared to the normal animals, indicating signs of injury in the myocardium. Treatment with curcumin significantly attenuated (*p* < 0.05) the oxidative stress as compared to HFD + STZ + ISO-treated mice. An increased lipid peroxidation (*p* < 0.001) was noticed in the myocardium of HFD + STZ + ISO-treated mice as compared to the normal group. Curcumin (200 mg/kg) significantly inhibited (*p* < 0.001) lipid peroxidation compared to that of the HFD + STZ + ISO-treated animals. However, AM630 pre-treatment inhibited the activity of curcumin as evidenced by the increase in oxidative stress (Figure 4).

### 3.6. Effect of Curcumin on Inflammatory Cytokines in HFD Fed Myocardial Infarcted Diabetic Mice

The levels of cytokines were determined so as to identify the inflammatory mechanism in the ISO-induced cardiotoxicity in diabetic animals. The contents of pro-inflammatory cytokines viz., TNF-α, IL-1β and IL-6 was significantly higher (*p* < 0.001) in the HFD + STZ + ISO group than in normal animals. A higher dose of curcumin (200 mg/kg) significantly lowered (*p* < 0.01) the expression of cytokines. However, pre-treatment with AM630 attenuated the effect of curcumin, therefore suggesting the involvement of CB2R (Table 2).

### 3.7. Effect of Curcumin on Histopathology of Myocardium in HFD Fed Myocardial Infarcted Diabetic Mice

**Figure 5:**
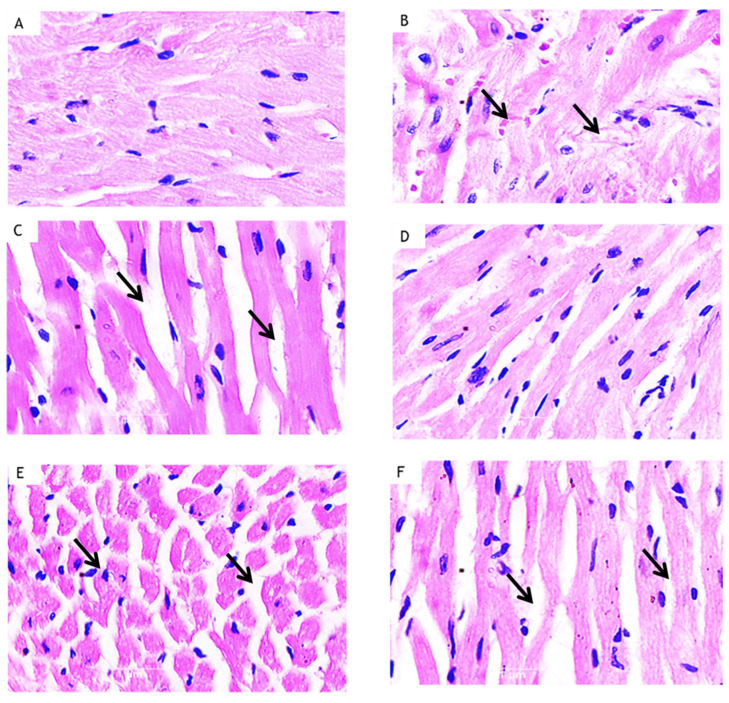
Effect of curcumin on the heart tissue in HFD-fed myocardial infarcted diabetic mice. (**A**) Light micrograph of normal group showing myocardial fibers are normal in structure and architecture. (**B**) HFD + STZ + ISO administered mice heart showing focal confluent necrosis of muscle fiber with inflammatory cell infiltration, oedema with fibroblastic proliferation along with extravasation of erythrocytes. (**C**) HFD + STZ + ISO + Cur (100) showing less myocardial injury, less oedema and inflammatory cells. (**D**) HFD + STZ + ISO + Cur (200) mice heart tissue showing mild oedema but without any signs of infarction. The myocardial fibers are normal in architecture. (**E**) HFD + STZ + ISO + Cur (200) + AM (1) showing marked myocardial necrosis, oedema with inflammatory cells infiltration. (**F**) HFD + STZ + ISO + AM (1) showing myocardial injury, oedema and inflammatory cells. The arrows indicate pathological changes in the myocardium.

The ultrastructural changes were captured with light microscopy. The normal group (A) showed normal myocardial fibers. On the other hand, the HFD + STZ + ISO-treated group (B) presented focal confluent necrosis muscle fiber with an infiltration of inflammatory cells, oedema with fibroblastic proliferation and the extravasation of erythrocytes. The HFD + STZ + ISO + Curcumin (100 mg/kg) group (C) revealed less myocardial damage, minimal oedema and the presence of inflammatory cells. Tissue with mild oedema but no infarction was observed in the HFD + STZ + ISO + Curcumin (200 mg/kg) group (D). However, the AM630 pre-treatment attenuated the effect of curcumin (200 mg/kg) (E), as evidenced by significant myocardial necrosis and oedema with an infiltration of the inflammatory cells. The AM630 treatment in the HFD + STZ + ISO group (F) showed intermediate myocardial damage, necrosis and the presence of inflammatory cells (Figure 5).

### 3.8. Molecular Docking

**Figure 6:**
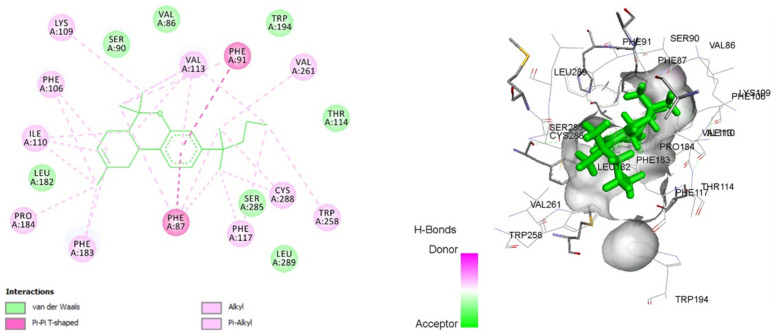
Two-dimensional and 3D interaction of JWH-133 with cannabinoid type-2 receptor (6KPC).

**Figure 7:**
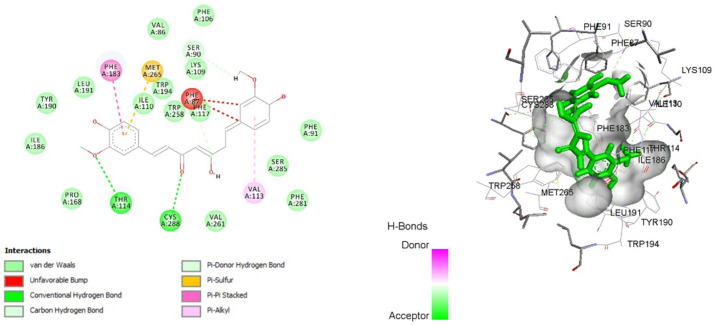
Two-dimensional and 3D interaction of curcumin with cannabinoid type-2 receptor (6KPC).

In this analysis, we compared the findings with the potent selective CB2R agonist JWH-133 for the docking of curcumin. The binding data suggest that curcumin has a good affinity towards CB2R with a binding energy of −12.8272 kcal/mol. A residual interaction analysis between the agonist and target receptor at the active binding site revealed that the JWH-133 bind with CB2R due to van der Waals interaction, Pi-Pi T-shaped interaction, alkyl, and Pi-alkyl interaction (Figure 6). Out of the 19 amino acid residues that interact with JWH-133, 13 were found to be common in curcumin (Table 3). Curcumin binds with CB2R by van der Waals interaction, a conventional hydrogen bond, carbon–hydrogen bond interaction, Pi–donor hydrogen bond interaction, Pi–sulfur interaction, Pi–Pi stacked interaction, and Pi–alkyl interaction (Figure 7). These interaction patterns give a strong impression that curcumin possesses good agonistic potential against CB2R.

Curcumin presents a comparable binding energy towards CB2R in the same way as JWH-133. Additionally, out of the 19 amino acid residues that interact with JWH-133, 13 were found to be in common with curcumin. Grid Parameters (Argus Lab.): Spacing 0.4 Å and Grid size 37.5236X Å, 22.4981Y Å and 20.6868Z Å.

## 4. Discussion

Molecular docking is a computational chemistry method that is commonly used as a successful tool for evaluating ligand–receptor interactions and for rational drug design. This approach has also been used along with in vivo studies that aim to confirm the ligand–receptor interaction [21,22]. We used a molecular docking study to evaluate the interaction of curcumin with CB2R before initiating in vivo studies because the affinity of curcumin at CB2R was not reported. An assessment of this interaction was essential because curcumin is reported to improve the systolic dysfunction and to prevent cardiac remodeling after MI [14], and myocardial CB2Rs are involved in the cardioprotective actions of endocannabinoids [7,10].

We observed an agonistic potential of curcumin at CB2R in an in silico molecular docking study. Therefore, we further evaluated the involvement of CB2R in the ameliorative effects of curcumin against MI in HFD-fed diabetic mice. We employed AM630 as a CB2R antagonist [23] to confirm the involvement of CB2R in the cardioprotective activity of curcumin. The in vivo experiments confirmed the ligand–receptor (curcumin-CB2R) interaction that was observed in the docking study. Although curcumin was found to protect myocardium from ISO-induced injury, pre-treatment with AM630 attenuated the cardioprotective action of curcumin. Viewed collectively, the data of the molecular docking screening and in vivo experiments suggest that CB2R plays a pivotal role in curcumin-triggered cardioprotection.

The outcomes of the present study further suggest the cardioprotective ability of curcumin, which occur by restoring several endogenous antioxidant components and an inflammation of the myocardial cells [14]. Furthermore, curcumin exhibited multiple activities such as control of blood glucose and lipids levels along with a transient conservation of the hemodynamic patterns, and the preservation of the myofibril structure and morphology.

The deposition of excess body fat leads to obesity, diabetes, atherosclerosis and several other metabolic disorders. We utilized the HFD and STZ treatment to simulate the diabetic state in mice, similar to in a clinical condition [24,25,26,27]. A significant weight gain was observed in mice kept on HFD compared to the chow diet-fed animals, although curcumin treatment led to the maintenance of body weight even in the presence of HFD [28]. Diabetic patients often suffer from some common symptoms viz., polyuria, polydipsia, polyphagia and fatigue. The administration of STZ in mice shared these symptoms with a notable boost in the food intake pattern throughout the study protocol [29].

Several studies suggest a stimulation of CB2R in immune and activated endothelial cells leading to anti-atherogenic and anti-fibrotic activity, and exert beneficial effects on cardiomyocytes. In this study, ISO successfully disturbed the ECG waveform as evidenced by ST segment elevation and a height increase, which indicates myocardial injury. Curcumin treatment effectively managed the ECG waveform and ST height elevation in diseased mice via CB2R activation in heart tissue. It is worth noting that the CB2R agonist effectively manages the acute inflammatory tissue injury related to MI [30]. The cardiac membrane is prone to insult the creatine kinase and dehydrogenase from the tissue as a cardiac injury marker into the bloodstream. Therefore, an increased content of these enzymes indicates myocardial necrosis. In this study, the contents of CK-MB and LDH (cardiac injury markers) were restored significantly by curcumin.

Recent reports confirmed that CB2R is present in human and rodent pancreatic β-cells and that the activation of CB2R improved insulin secretion and glucose metabolism [31]. The administration of curcumin (200 mg/kg) significantly improved pancreatic β-cell functions and the systemic secretion of insulin as evidenced by a decrease in the glucose level. HFD is known to follow the pattern of a sedentary lifestyle, and hence is liable to induce the level of TC, TG, and LDL-C, and downgrade HDL-C. The present study supports and is in-line with an increase in the lipid profile as evidenced in the diseased group. Treatment with curcumin restored the levels of the different lipids.

Previous findings suggests that patients with hepatic steatosis and steatohepatitis present an overexpression of CB2R in hepatocytes, cholangiocytes, and stellate cells of the liver. Furthermore, CB2R overexpression in non-alcoholic fatty liver disease indicates its role in related diseases such as obesity, type 2 diabetes, and hypertriglyceridemia [32]. In the present study, curcumin actively maintained the levels of liver enzymes such as SGOT and SGPT, thereby signifying the hepatoprotective role of curcumin via CB2R modulation. A deregulated redox homeostasis triggers the overproduction of ROS and lipid peroxidation, which consequently results in cellular injury, particularly in highly perfused tissue such as myocardium [33]. It has been emphasized that oxidative stress and lipid peroxidation are common factors evident in the etiopathogenesis of various disorders.

MDA, a widely accepted indicator of lipid peroxidation is generally produced by polyunsaturated fatty acids (PUFA) of the membrane. The ROS react with PUFA and trigger lipid peroxidation [34]. The increased level of MDA indicates an excessive formation of free radicles and results in irreversible damage to the heart. A mutually supportive enzyme system consisting of SOD, CAT, and GSH serves as the primary defense mechanism that protects from oxidative damage [35].

In this study, these supportive enzymes increased their presence and diminish the generation of lipid peroxidation, thereby supporting the role of CB2R agonist curcumin in preserving the antioxidant status. The role of CB2R in the regulation of pro-inflammatory cytokines has been researched extensively. The results of the present study confirm the effects of curcumin as an exogenous CB2R agonist, and a pharmacologic role for CB2R on ISO-induced MI in HFD-fed diabetic mice. Curcumin treatment demonstrated a depletion in the levels of inflammatory mediators, therefore suggesting an anti-inflammatory role of curcumin via CB2R activation.

Curcumin administration in ISO-treated diabetic mice led to the protection of normal morphology of the myocardium without noticeable signs of infarction. Conversely, the mice treated with AM630 showed an increase in myocardial necrosis, oedema and increased the level of inflammatory cells. This indicates that curcumin activates CB2R in cardiomyocytes and protects them against ISO-induced injury in HFD-fed diabetic animals. A similar cardioprotective effect of curcumin was reported in earlier studies [36,37]. In addition to its direct action on the myocardium, curcumin showed a cardioprotective effect against HFD-induced atherosclerosis [38,39]. These data suggest that the protective effect of curcumin might be associated with its direct action on cardiomyocytes as well as its anti-atherosclerotic activity.

Taken together, curcumin treatment offers protection against MI in diabetic conditions and the mechanism for this favorable effect could be partially attributed to its CB2R-activating property. Thus, the present study provides direct evidence that CB2R activation downregulates oxidative stress and subsequent tissue injury as well as regulating various metabolic markers. This study, for the first time, demonstrates curcumin to be a CB2R agonist that contributes to the cardioprotective responses against the HFD fed ISO-induced MI in diabetic mice.

## 5. Conclusions

The present study demonstrates the cardioprotective effect of curcumin through CB2R activation in myocardial infarcted diabetic mice. However, additional molecular studies are required to understand the underlying signaling pathways in the ameliorative actions of curcumin.

## Figures and Tables

**Table 1:** Effect of curcumin, alone and in combination with AM630 on blood glucose and lipid profile in HFD-fed myocardial infarcted diabetic mice.

Treatments	Blood Glucose (mg/dL)	TC (mg/dL)	TG (mg/dL)	LDL–C (mg/dL)	HDL–C (mg/dL)
**Normal**	67.7 ± 5.80	113 ± 1.32	75.0 ± 2.23	29.9 ± 2.30	78.7 ± 2.51
**HFD + STZ + ISO**	288 ± 5.68 ^###^	197 ± 2.83 ^###^	188 ± 3.91 ^###^	68.2 ± 1.95 ^###^	50.4 ± 4.84 ^###^
**HFD + STZ + ISO + Cur (100)**	164 ± 3.26 *	161 ± 2.09 **	132 ± 14.9 ***	30.1 ± 8.25 **	70.1 ± 2.24 ***
**HFD + STZ + ISO + Cur (200)**	121 ± 7.49 **	121 ± 2.20 ***	85.5 ± 4.27 ***	31.4 ± 4.34 **	78.4 ± 2.58 ***
**HFD + STZ + ISO + AM (1) + Cur (200)**	257 ± 4.85	228 ± 12.5	173 ± 4.63	67.6 ± 9.47	49.4 ± 1.84
**HFD + STZ + ISO + AM (1)**	215 ± 4.12	177 ± 6.67	139 ± 10.21	56.7 ± 10.03	44.3 ± 2.50

The data were expressed as mean ± SEM, and analyzed by one-way ANOVA followed by Bonferroni’s post hoc test. ^###^ *p* < 0.001 vs. normal; * *p* < 0.05, ** *p* < 0.01, *** *p* < 0.001 vs. HFD + STZ + ISO. AM: AM630; Cur: Curcumin; HDL-C: High density lipoprotein-Cholesterol; HFD: High-fat diet; ISO: Isoproterenol; LDL-C: Low density lipoprotein-Cholesterol; STZ: Streptozotocin; TC: Total cholesterol; TG: Triglycerides.

**Table 2:** Effect of curcumin alone and in combination with AM630 on the levels pro-inflammatory cytokines in HFD-fed myocardial infarcted diabetic mice.

Treatments	TNF-α (pg/mL)	IL-1β (pg/mL)	IL-6 (pg/mL)
**Normal**	21.0 ± 1.20	182 ± 8.01	35.3 ± 3.99
**HFD + STZ + ISO**	34.4 ± 3.24 ^###^	369 ± 3.50 ^###^	66.4 ± 3.27 ^###^
**HFD + STZ + ISO + Cur (100)**	21.8 ± 2.09 **	197 ± 1.18 **	39.7 ± 1.08 *
**HFD + STZ + ISO + Cur (200)**	17.6 ± 1.81 ***	177 ± 6.72 ***	26.7 ± 1.65 ***
**HFD + STZ + ISO + AM (1) + Cur (200)**	27.4 ± 1.35	282 ± 3.22	65.4 ± 9.20
**HFD + STZ + ISO + AM (1)**	29.1 ± 0.95	284 ± 4.69	50.8 ± 8.66

The data were expressed as mean ± SEM, and analyzed by one-way ANOVA followed by Bonferroni’s post hoc test. ^###^ *p* < 0.001 vs. normal; * *p* < 0.05, ** *p* < 0.01, *** *p* < 0.001 vs. HFD + STZ + ISO. AM: AM630; Cur: Curcumin; HFD: High-fat diet; IL-1β: Interleukin-1 beta; IL-6: Interleukin-6; ISO: Isoproterenol; STZ: Streptozotocin; TNF-α: Tumor necrosis factor-alpha.

**Table 3:** Comparative evaluation of affinity of curcumin vs. standard agent, JWH-133 for CB2R (6KPC).

Compound ID (s)	Binding Energy (kcal/mol)	Enzyme’s Binding Site Residue
JWH-133	−14.8967	VAL 86, **PHE 87**, **SER 90**, **PHE 91**, PHE 106, **LYS 109**, **ILE 110**, **VAL 113**, **THR 114**, **PHE 117**, LEU 182, **PHE 183**, PRO 184, **TRP 194**, **TRP 258**, VAL 261, **SER 285**, **CYS 288**, LEU 289
Curcumin	−12.8272	**PHE 87**, **SER 90**, **PHE 91**, **LYS 109**, **ILE 110**, **VAL 113**, **THR 114**, **PHE 117**, **PHE 183**, ILE 186, TYR 190, LEU 191, **TRP 194**, **TRP 258**, MET 265, **SER 285**, **CYS 288**

## Data Availability

Not applicable.
